# Neural pathways of maternal responding: systematic review and meta-analysis

**DOI:** 10.1007/s00737-018-0878-2

**Published:** 2018-07-09

**Authors:** Sarika Paul, Josie Austin, Rebecca Elliott, Ian Ellison-Wright, Ming Wai Wan, Richard Drake, Darragh Downey, Alya Elmadih, Ipshita Mukherjee, Lisa Heaney, Steve Williams, Kathryn M. Abel

**Affiliations:** 1 Division of Diabetes, Endocrinology and Gastroenterology, University of Manchester, Manchester, UK; 2 Centre for Women’s Mental Health, University of Manchester, Manchester, UK; 3 School of Health Sciences, University of Manchester, Manchester, UK; 4 Neuroscience and Psychiatry Unit, University of Manchester, Manchester, UK; 5 Avon and Wiltshire Mental Health Partnership NHS Trust, Manchester, UK; 6 Manchester Academic Health Science Centre (MAHSC), University of Manchester, Manchester, UK; 7 Greater Manchester Mental Health NHS Trust, Manchester, UK; 8 Pennine Acute Hospital NHS Trust, Manchester, UK

**Keywords:** Maternal sensitivity, Oxytocin, Mother-infant, fMRI

## Abstract

Functional magnetic resonance imaging (fMRI) has increasingly been employed to establish whether there is a specific brain neural network dedicated to maternal responsiveness. We undertook systematic review and meta-analysis of all studies in which healthy new mothers were exposed to visual stimuli of own versus other infants to determine the quality of evidence for a dedicated maternal neural network. Systematic literature review revealed a pattern of specific neural responses commonly induced by visual infant paradigms. Brain areas consistently reported as activated in mothers in response to own versus unknown infant included the left thalamus, bilateral pre-central gyrus, left limbic lobe, uncus, amygdala and left caudate. These regions are implicated in reward, attention, emotion processing and other core social cognitive skills. Meta-analysis, however, revealed a more limited subset of brain areas activated in mothers specifically in response to their own versus unknown infant and suggested considerable inter-study variability. Further work is needed if functional imaging is to become an objective tool for the assessment of neural pathways associated with distinct patterns of maternal care behaviour. Such a tool would be invaluable in developing biomarkers of neural activity associated with healthy maternal care and for monitoring treatment/intervention effects of costly parenting interventions.

**Electronic supplementary material:**

The online version of this article (10.1007/s00737-018-0878-2) contains supplementary material, which is available to authorized users.

## Introduction

Since the first functional magnetic resonance imaging (fMRI) study of seven new mothers listening to sounds of infants crying nearly two decades ago (Lorberbaum et al. 1999), this powerful technique has been increasingly employed in an attempt to identify a specific brain circuitry for maternal care behaviour (Swain et al. 2014). Using the blood oxygenation level-dependent (BOLD) effect, fMRI can indicate which brain areas are being used in response to different conditions—this works on the principle that areas which are more active in response to a particular stimuli will require greater perfusion to meet the demand for oxygen. Squire and Stein (2003) argued that use of fMRI (with a spatial resolution ~ 3–4 mm; Glover 2011) could strengthen our understanding of maternal care behaviour in order to provide much needed insights into its neural basis.

‘Maternal sensitivity’ as a construct that involves ability for perception, accurate interpretation and appropriate responsiveness to infant’s signal (Ainsworth et al. 1978) is believed to foster secure attachment and promote child’s development. Literature has suggested that variation in maternal sensitivity (i.e. sensitive and less sensitive mothers) is an outcome of interaction between behavioural, social factors that charts a discrete profile of the maternal brain that is mediated by stress- and reward-related neural systems.

In imaging terms, ‘maternal responsiveness’ refers to neural brain activation patterns associated with a mother’s response to own infant stimuli. Within clinical settings, observations of maternal-infant interactions by professionals provide useful ‘snapshots’ that may be helpful in highlighting specific issues requiring extra support. However, due to their very nature, these judgements are quite informal and subjective. There are standardised and validated observer-rated scales of maternal or caregiver interaction available (e.g. CARE-index, Crittenden 1979–2004). Such scales, however, are time-consuming and costly requiring trained raters and so are only used in research settings. fMRI offers the opportunity to develop newer more accurate observer-rated scales of maternal interaction that can be used in clinical settings or to evaluate innovative parenting interventions.

It is likely that a number of different pathways or mechanisms could result in a mother exhibiting suboptimal maternal care. Studies that focus on the neurobiological correlates of maternal sensitivity in a distinct group of mothers representing natural variations in maternal sensitivity were few, including one of our own (Elmadih et al. 2016). Preliminary evidence from these studies showed that maternal sensitivity appears to be accompanied by a discrete neural correlate and that modulation of maternal brain responses by oxytocin (OT) is possible (i.e. as evidenced by the significant correlation between plasma OT and BOLD activation). Yet, further studies are needed before fMRI could be used as a biomarker for maternal sensitivity. Other analysis parameters, such as connectivity, are being explored to see if more subtle changes are present. In the future, such parameters could be used in conjunction with reported and observational measures to help identify mothers at risk of suboptimal responsiveness at an earlier stage and help to evaluate the efficacy of interventions designed to enhance sensitive parental caregiving.

Previous fMRI studies have used either auditory (infant cries) or visual (images/videos of infants) stimuli to compare maternal brain responses to infant versus non-infant control stimuli; or to own versus unknown infant control stimuli. Such studies have used a range of sample sizes from small to large numbers of participants (*N* = 4–30).

**Fig. 1:**
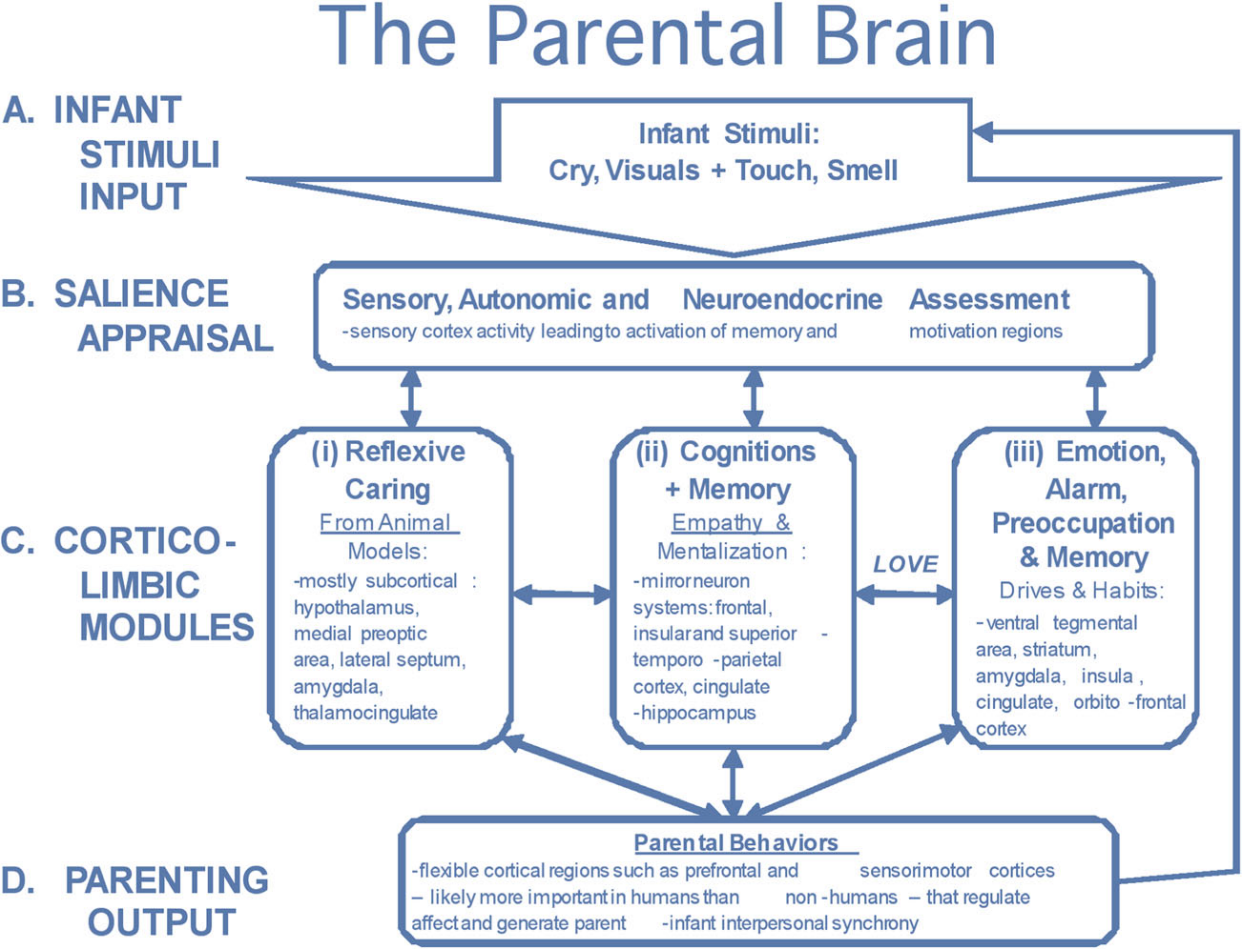
Swain’s model for the brain basis of parental responsiveness. Source: Swain (2010); used with the permission of the author

Studies reveal a diverse pattern of responses to infant stimuli, including activation in dopamine-associated reward processing areas, cortico-limbic modules, emotional processing pathways and the mirror neuron system. Swain (2010) proposed a model for the brain circuitry underlying parental responsiveness involving subcortical and cortical regions (see Fig. 1). This model has been updated (Swain et al. 2014) with a breakdown of activate areas commonly reported by parental imaging studies in response to infant stimuli.

Following salience appraisal involving sensory, autonomic and neuroendocrine pathways which activate the sensory cortex, three potential responses were identified:

1. ‘Reflexive caring’, located in the subcortical brain, including the hypothalamus, medial preoptic area, lateral septum, amygdala and thalamocingulate regions;
2. ‘Empathic and related cognitive emotional responses associated with mentalization’, including mirror neuron systems in the frontal, insular and superior temporoparietal cortices, cingulate and regions associated with memory such as the hippocampus;
3. ‘Drives and habits related to emotions, alarm and preoccupations’, including the ventral tegmental area, striatum, amygdala, insula, cingulate and orbitofrontal cortex.

This model is based on a review of neuroimaging studies to date. Our meta-analysis seeks to refine this parental brain model using quantitative techniques. Given the diversity of findings, spatial meta-analysis (Turkeltaub et al. 2002) may be the most appropriate way more robustly to establish whether (and which) specific brain areas are most likely to represent a dedicated neural network for maternal responsiveness. Lieberman and Cunningham (2009) recommended greater focus on drawing conclusions from meta-analysis rather than from a single fMRI study. This has the potential to provide more reliable results by reducing type I errors (i.e. false positives) and, where appropriate, allowing for more lenient thresholding to avoid type II errors (i.e. missing true effects).

The primary aim of this study, therefore, was to determine whether there is indeed evidence for a dedicated maternal neural network associated with brain responses to visual cues from own versus control infants (up to the age of 2 years). First, we undertook a systematic review to summarise reported maternal brain activation. Second, we examined quantitatively evidence for distinct activation patterns in maternal responding to own infant, focusing on studies using visual stimuli. Auditory stimuli used in studies of maternal response typically comprise emotionally laden baby laughter or cry sounds. By contrast, studies using visual stimuli include neutral as well as emotional faces, allowing responses to infants per se to be disambiguated from responses specifically to infants expressing emotion. It is important to note that whilst visual and auditory cues are most commonly used within fMRI imaging studies due to the constraints of the technique, in a naturalistic setting, other infant stimuli such as tactile and olfactory are likely to influence maternal brain responsiveness.

We hypothesised that (1) maternal brain responsiveness to infant versus control stimuli in healthy new mothers is associated with a distributed neural network; (2) this network is consistent with current models of maternal neural responses (Swain et al. 2014); (3) differences in activation to own versus control infant will be particularly significant for neural regions associated with hedonic responses (reward from the experience of maternal love) and emotional salience. More specifically, we predict that there will be differences in activation in the thalamus, hypothalamus, and septal regions, orbitofrontal cortex, amygdala, insula and basal ganglia (in relation to the hedonic response and emotional salience of infant stimuli).

## Methods

### Systematic review: inclusion criteria for studies

Only original fMRI studies were included, in which healthy mothers were exposed to visual stimuli of own as well as control infants (up to 24 months in age), and a comparison of maternal brain activation in response to these stimuli was undertaken. To reduce bias in spatial meta-analyses, it is important that only data from independent studies is included. Therefore, study data were excluded if it contributed to another study, in which case the study with the largest group size was included. Studies including infants older than 24 months were eliminated to avoid excess heterogeneity between infant stimuli that may minimise the areas of brain activation identified through meta-analysis.

### Identification of studies

Papers were identified by typing the word combination [‘parent’ or ‘mother’ or ‘maternal’] and [‘fMRI’ or ‘imaging’] into the search engines ‘PubMed’, ‘Web of Knowledge’ and ‘Google Scholar’. The words ‘fMRI’/’imaging’ and ‘parent’/‘mother’/‘maternal’ had to appear in the title or abstract in order for the papers to be identified. Works that incorporated one of these words, such as ‘mother*ing*,’ were also valid. Relevant research and review papers were searched for mention of additional studies. Researchers identified as leading the field were contacted to enquire whether they had carried out additional research. The methodology was in line with the principles of systematic reviews as outlined by the Cochrane Handbook for Systematic Reviews of Interventions (2011).

### Method of meta-analysis

Estimates of association were meta-analysed using the software GingerALE (Eickhoff et al. 2009), which is suitable for studies with comparable experimental paradigms even if the number of studies is small. Studies using photographs or videos of infants were combined in the analysis. Confirmation was obtained from studies’ authors that data did not overlap between studies to ensure data points were not included more than once. All reported findings satisfy criteria of *p* ≤ 0.001 for fixed effects (whole brain uncorrected) or *p* ≤ 0.05 FWE corrected/*p* ≤ 0.001 whole brain uncorrected for random effects analysis. The field strength of the scanners used in each included study was also recorded (see supplementary Tables 1–3). Potentially, studies using lower field strength scanners may impact the spatial resolution but not localisation.

## Results

### Identified studies

Twelve identified studies were published between November 2002 and January 2016. Results from an additional unpublished study (Abel et al. 2018) were obtained directly from the researchers. Strathearn et al. (2005) was reported to be an earlier version of the authors’ later papers (Strathearn et al. 2008, 2009), and was hence excluded from reported summaries and further analyses leaving. Of the 12 studies remaining, 7 used static images (i.e. photographs) and 5 used moving images (i.e. videos).

Supplementary Table 1 (S1) provides a chronological summary of all identified studies (both static images and videos), the stimuli used, their research design, and the contrasts they provided

### Summary of identified brain activity

An overview of areas reported as significantly activated in identified studies of mothers exposed to visual stimuli of own versus control infant stimuli is given in supplementary Tables 2 and 3 (S2 and S3). The diversity of reported results suggests that a broad range of brain areas are associated with a mother responding to visual stimuli of her own infant. Within the current literature, there is a wide range of findings from studies with relatively small sample sizes. Meta-analysis was therefore performed and related to existing literature.

### Studies included in the meta-analysis

Table 1 shows a summary of included and excluded studies, how many participants took part in each study, and how many relevant foci were provided by each study. Of the 12 identified studies, 9 (Nitschke et al. 2004; Ranote et al. 2004; Noriuchi et al. 2008; Lenzi et al. 2009; Strathearn et al. 2008; Atzil et al. 2011; Barrett et al. 2012; Wan et al. 2014; Abel et al. 2018) provided foci for maternal brain activation in response to images of own versus control children (familiar or unknown). Two studies (Swain et al. 2003; Strathearn et al. 2009) were excluded as it was not possible to obtain the relevant foci for them—they were not reported in the papers and the authors were unable to provide them upon request.

**Table 1:** Included and excluded studies

Study	Included/excluded	Reason for exclusion	Number of participants	Number of reported foci
Strathearn and McClure (2002)	Included	N/A	8	9
Swain et al. (2003)	Excluded	Unable to obtain foci	9–14	N/A
Nitschke et al. (2004)	Included	N/A	6	6
Ranote et al. (2004)	Included	N/A	10	3
Noriuchi et al. (2008)	Excluded	Small volume correction applied	13	N/A
Lenzi et al. (2009)	Included	N/A	16	7
Strathearn et al. (2008)	Included	N/A	28	50
Strathearn et al. (2009)	Excluded	Unable to obtain relevant foci and study not independent from Strathearn et al. (2008)	30	N/A
Atzil et al. (2011)	Included	N/A	23	21
Barrett et al. (2012)	Included	N/A	22	14
Wan et al. (2014)	Included	N/A	20	20
Abel et al. (2018)	Included	N/A	14	6
Overall	12 studies identified (9 studies included)	3 studies excluded	147 participants included	136 foci included (from 9 studies)

One study (Atzil et al. 2011) reported foci for both intrusive and synchronous mothers; however, for the purpose of the analyses, foci for the combined results were used (i.e., including all mothers). In this study, synchronous mothers were those whose behaviour was appropriate and relevant to the infant signals whilst intrusive mothers demonstrated excessive maternal behaviour not in keeping with the infants’ signals. Another study (Strathearn et al. 2008) reported foci for activation in response to happy, sad and neutral infant faces, while Barrett et al. (2012) reported foci using happy and sad infant faces; for the purpose of the analysis, the combined results (i.e. responses to all faces) were used. One study applied small volume correction to their initial results (Noriuchi et al. 2008)—it was not possible to obtain the coordinates for activation prior to small volume correction and so this study was excluded in order to prevent spatial bias. The nine included studies provided 136 foci overall, with 147 subjects included. Eight studies provided coordinates in Talairach space, while one study (Abel et al. 2018) reported coordinates in MNI space. These were converted into Talairach space using GingerALE.

Probability maps were generated uncorrected at *p* < 0.0001. This strict *p* value was chosen in lieu of correction for multiple comparisons and in absence of cluster level stats. This approach is recommended in the GingerALE manual. Once the analysis had been run in GingerALE, ‘Mango’ software (v 3.8; Designed and developed by Jack L. Lancaster, Ph.D. and Michael J. Martinez) was used to produce images of activated brain areas.

### Results of meta-analysis

**Fig. 2:**
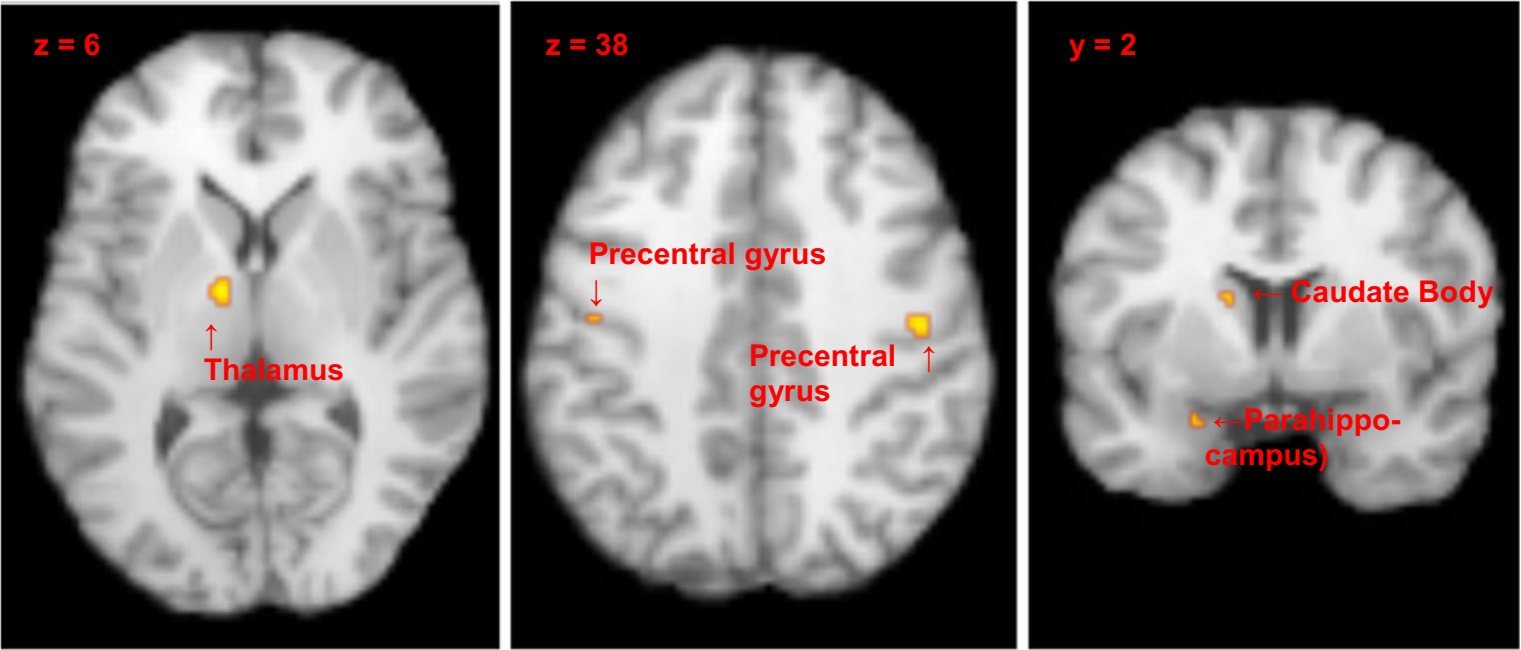
Brain activation in mothers exposed to visual stimuli for own versus control infant. Activation was found within the thalamus, post-central gyri, caudate and parahippocampus. *P* = 0.0001, uncorrected

Table 2 shows results for meta-analysis of nine studies (total sample = 147 participants), uncorrected (*p* = 0.0001) level of significance. Based on the combined results of these studies, maternal exposure to visual stimuli of own versus control children was associated with activation (uncorrected *p* = 0.0001) of the following brain areas: left thalamic ventral lateral nucleus, bilateral precentral gyrus (BA 4), left uncus (BA 34) and amygdala in the limbic lobe and left caudate body (Fig 2).

**Table 2:** Summary of maternal brain activity as identified by meta-analyses in response to visual stimuli of own and control infant (*p* = 0.0001) (*n* = 147)

	Left	Right
Septal regions (MPOA/VBNST/caudate head)	ACT	
Midbrain		
Hypothalamus		
Thalamus	ACT	
Limbic structures		
Amygdala	ACT	
Anterior cingulate		
Middle cingulate		
Posterior cingulate		
Hippocampus		
Basal ganglia		
Striatum/putamen/nucleus accumbens		
Lentiform nucleus/globus pallidus		
Insula		
Frontal cortex		
Orbitofrontal/inferior frontal gyrus		
Medial/middle frontal gyrus		
Ventral prefrontal cortex		
Superior frontal gyrus		
Precentral gyrus	ACT	ACT
Dorsolateral prefrontal cortex		
Temporal/parietal cortex:		
Temperoparietal cortex		
Fusiform gyrus		
Temporal/auditory cortex		
Parahippocampal/limbic lobe	ACT	
Occipital cortex		
Cerebellum		

*ACT* activated

## Discussion

To our knowledge, this is the first time formal meta-analysis methodologies have been used to integrate evidence for a distributed maternal brain circuitry. The main finding was that significant activation occurred in far fewer areas than suggested by conventional systematic literature review. Meta-analysis identified increased activation in response to own versus unknown infant visual stimuli in left thalamic ventral lateral nucleus; bilateral precentral gyrus (BA 4), left uncus (BA 34) and amygdala in the limbic lobe, and left caudate body.

Our findings differ from models previously proposed by Swain (2008), Swain et al. (2014) primarily because we highlight fewer areas robustly activated by visual contrasts of own versus unknown infants.

Although the diversity of brain areas robustly activated in response to infant visual stimuli concords with the complexity of maternal behaviour, it is also likely to reflect differences in the stimuli and fMRI paradigm across studies, as well as the variety of neural responses which depend on the particular ‘affective cognitive’ style of individual mothers. The interface between affective and cognitive processing, termed ‘affective cognition’, includes the ability to attend to, recognise and interpret different emotional stimuli (e.g. emotive sounds, words, facial expressions, body language), as well as how such information is categorised, encoded and subsequently recalled from memory to guide current behaviour (Elliott et al. 2011). Thus, a mothers’ individual affective cognitive style is likely to be another factor influencing the neural responses identified by fMRI. We consider, therefore, evidence from fMRI studies of affect, emotion and cognitive processing that should (in relation to the each of the brain areas) be activated robustly following meta-analysis.

### Thalamus

We identified thalamic activation in response to own versus control infants; this is consistent with previous studies in mothers who reported an increase of positive emotional experience when seeing their own infants (Swain et al. 2005). The thalamus is high in dopaminergic projections (Sánchez-González et al. 2005) and activation within this area has been associated with motivational salience of infant stimuli (Swain et al. 2014).

Thalamic activation is found in neural responses to own versus other infants in both visual and infant cry paradigms (Swain et al. 2014); and it is activated differently by infant compared to adult, or animal faces of any age (Caria et al. 2012). In the Swain et al. models (2014) (Swain 2008), the thalamus is considered important for reflexive caring. However, thalamic activation may also be part of a thalamic-cortical-basal ganglia loop associated with repetitive or reflex behaviours (Baxter 2003; Leckman et al. 2004).

### Precentral gyrus

Our analysis revealed bilateral activation in precentral gyrus. Human maternal care is a very tactile and active process which involves the coordination of many motor outputs; lifting, cuddling, cradling, rocking, feeding etc. Activity in this area may, therefore, be associated with planning behavioural outputs, provoked in a mother when she views images of her infant and associated with her intent or desire to respond to her child needs (Swain et al. 2014). Alternatively, this activity may be related to the ‘readiness-to-act’, perhaps associated with hypervigilance that comes with appropriate maternal sensitivity.

### Limbic lobe

Left limbic lobe activation, comprising activation in uncus (BA 34) and amygdala occurred in mothers looking at own versus other infants. The uncus is the most anterior and medial portion of the parahippocampal gyrus, located directly above the amygdala (Tamraz et al. 2004). The attribution of emotional salience to stimuli engages these areas along with insula, amygdala and basal ganglia (Phan et al. 2004).

The parahippocampal lobe plays an important role in encoding emotional memory and retrieval (Ferreira et al. 2003). The ability to call on previous experience is an essential aspect of maternal caregiving, especially in new mothers who are learning new skills constantly. Processing and regulation of emotional information, alongside memory retrieval, is likely to be a key aspect in appropriate maternal behaviour. In Swain et al.’s 2014 model, several other areas including the insula, cingulate and orbitofrontal cortex were included under the ‘drives and habits’ response. Survival of the limbic lobe’s focus of activation through meta-analysis shows that it is consistently activated by maternal imaging paradigms. The absence of foci of activation within other limbic areas such as the insula and cingulate cortices may reflect the diversity of paradigms in studies of maternal responses to infant stimuli. For example, in Swain et al.’s 2014 review, children aged up to 6 years, fathers and non-visual stimuli have been included.

### Caudate

Our analysis revealed a focus of activation within the left caudate nucleus, one of the structures of the basal ganglia. The basal ganglia are part of a reward network that includes septal regions and left orbitofrontal cortex (Bechara et al. 1994; Peters and Büchel 2010). Basal ganglia activation has also been associated with obsessional behaviours (Harsányi et al. 2007). It has been suggested that activation of these areas implies that a mother’s feelings towards her new infant are akin to obsessional or addictive behaviour in terms of their reward response (Burkett and Young 2012). This notion is supported by mothers’ descriptions of the powerful emotions they experience on exposure to their new infant (e.g., Leckman et al. 1999; Swain et al. 2005). The dopaminergic reward system in the midbrain is crucial for the motivation of caregiving in new mothers (Numan and Numan 1997; Strathearn 2011). Activation of basal ganglia in response to own versus control children mirrors findings of similar responses to romantic partners (Bartels and Zeki 2004), which the authors associated with the feeling of reward that comes from love and attachment for a salient other and with exposure to representations of that object of attachment.

### Strengths and limitations

As far as we are aware, this is the first study to use systematic review and meta-analysis to assess evidence for the neural correlates of maternal responding. Systematic review ensures that all studies meeting predefined inclusion criteria are included and are identified using an explicit search technique. This reduces selection bias when describing positive findings from the research literature. Meta-analysis provides a quantitative method for integrating findings with the advantages that (1) consistent regional effects are identified (and inconsistent ones discarded), (2) statistical power is increased and (3) use of coordinate data avoids the problems of different neuro-anatomical labelling systems which may be present in the primary studies.

Of the nine included studies, three included only first-time mothers (Nitschke et al. 2004; Lenzi et al. 2009; Strathearn et al. 2008). A limited literature has addressed the impact of ‘first time’ mothering on maternal brain responsiveness (e.g. Strathearn et al. 2008), maternal sensitivity (e.g. Elmadih et al. 2014) or oxytocin responsiveness (Feldman et al. 2010). However, no significant differences were found in these parameters in relation to first-time mothering, possibly because the lack of experience with parenting, seen in first-time mothers reflected as maternal ‘competency’ rather than maternal ‘sensitivity’.

There are some important limitations to consider. None of the included studies compared mothers’ responses to own infants with responses to other key attachment figures including romantic partners or other reward stimuli. It is not possible, therefore, to establish whether activation of the identified brain areas are unique to mothers being exposed to their infants. We do not believe that the quality of included studies caused the relative lack of brain areas showing significant activation following meta-analysis. It may be simply that even the relatively minor variations in paradigms between studies produced too much change in voxel-based foci of activation to generate meaningful results in meta-analysis. The GingerALE software advises that studies with similar paradigms can be used, but the use of a standardised maternal sensitivity paradigm may be of considerable value in generating more reproducible and robust results, as will be discussed below. Interpretation of patterns of activation may also be compromised because stimuli were only representations of children and, although some stimuli were videos which provide more ecological validity, the visual stimuli were presented without auditory (Swain and Lorberbaum 2008), olfactory (Porter et al. 1983) or tactile cues (Kaitz et al. 1992); all of which induce maternal neural responses. Although stimulus modalities are not regularly combined within studies, addition of such cues could add ecological validity to future research designs.

Most (but not all) of the included studies (Nitschke et al. 2004; Lenzi et al. 2009; Strathearn et al. 2008; Barrett et al. 2012; Wan et al. 2014; Abel et al. 2018) clearly specified using only right-handed mothers. This variation could potentially have impacted the interpretation of activation localisation for example in areas such as the precentral gyrus (although we found bilateral activation of this area)—future studies would benefit from clearly specifying handedness.

As in any meta-analysis, the studies with more participants may have greater effect on the final results, skewing them towards findings in those studies. Similarly, heterogeneity between samples, stimuli and designs may reduce areas of brain activation identified. We minimised this bias by eliminating studies with infants older than 24 months (the oldest infants in the included studies were 16 months) which provided an acceptably narrow range given the nature of meta-analysis requiring integration of individual paradigms. All studies used unknown children as control stimuli.

### Future directions

Our findings suggest that future imaging research in mothers should be collaborative. This would allow standardisation of experimental fMRI paradigms using a greater number and a more demographically diverse subject group (e.g. ethnicity or parity). This is key to increasing statistical power and robustness of the results (Dale 1999).

We also suggest comparisons with matched non-mothers, mothers with varying behavioural maternal sensitivity (Elmadih et al. 2014), mothers with mental illness (Abel et al. 2018) and fathers of the same infant, as piloted by Swain et al. (2003). Infant temperament and sociality is known to regulate and interact with maternal behaviour, but few studies have examined this in relation to maternal neural responses. Infant eye tracking could be employed alongside fMRI to explore such associations further. Multisite collaboration is also required to recruit mothers from hard-to-access groups (e.g. those with severe mental illness) which may be associated with poor maternal sensitivity so that we may optimise the patient and public benefits of this innovative research stream. Although much can be done to share fMRI stimuli and analysis paradigms, cross-country, intergroup funding applications are key to facilitate such an approach in the future.

## Conclusions

Functional imaging is increasingly employed to study the neurobiology underpinning human parental response. Regional brain activation may serve as a benchmark of healthy responsiveness. Research is beginning to detail ways in which brain responses to infants may be modulated in new parents. Such approaches are likely to be of increasing interest in the development of novel parenting interventions; for example, those involving the administration of intranasal oxytocin alone or in combination with behavioural approaches. However, its utility can only be exploited to improve clinical outcomes if studies produce consistent results and many studies have been too small and varied to do so. Meta-analysis provides a powerful technique whereby fMRI data can be integrated to identify robust changes. This study highlights the importance of using meta-analysis, as well as common experimental designs, to allow larger sample sizes.

## Electronic supplementary material

ESM 1 (DOC 196 kb)
