# Supplementary material for: Neural pathways of maternal responding: systematic review and meta-analysis
Source: Arch Womens Ment Health. 2018 Jul 9;22(2):179–87. doi: 10.1007/s00737-018-0878-2 (PMC6440933; doi:10.1007/s00737-018-0878-2)
Supplement: Supplementary file 1 — (DOC 196 kb) [file 737_2018_878_MOESM1_ESM.doc]

***Supplementary table 1***: Identified studies

| **Study** | **Type of stimuli** | **Study design** | | **Experimental stimuli** | **Control stimuli** | **Number of participants** | **Contrasts (own versus control child) reported** |
| --- | --- | --- | --- | --- | --- | --- | --- |
|
| **Strathearn & McClure (2002)** | Visual (static images) | 3T, 6s events | | Photographs of own infant (3 to 8 months old) | Photographs of unknown infants (3 to 8 months old) | 8 mothers (3 to 8 months postpartum) | Yes |
| **Swain et al. (2003)** | Visual (static images) | 3T, 30s blocks | | Photographs of own and unknown infant (2 weeks to 4 months old) | Images of houses, Photographs of unknown infants (2 weeks to 4 months old) | 9-14 mothers; 4-9 fathers (2 weeks to 4 months postpartum) | Yes |
| **Nitschke et al. (2004)** | Visual (static images) | 1.5T, 30s blocks | | Photographs of own infant (2 to 4 months old) | Photographs of unknown infants (2 to 4 months old) | 6 right-handed mothers (2 to 4 months postpartum) | Yes |
| **Strathearn et**  **al. (2005)** | Study excluded from further synthesis as it was an earlier version of Strathearn et al. (2008) and Strathearn et al. (2009). | | | | | | |
| **Lenzi et al. (2008)** | Visual (static images) | 3T, 2s mini blocks | Photographs of own child (6-12 months old; neutral, distressed, joyful and ambiguous expressions) | | Photographs of unknown children (6-12 months old; neutral, distressed, joyful and ambiguous expressions) | 16 right-handed mothers (6-12 months postpartum) | Yes |
| **Strathearn et al. (2008)** | Visual (static images) | 3T, 2s events | Photographs of own infant (5 to 10 months old; happy, neutral and sad expressions) | | Photographs of unknown infants (5 to 10 months old; happy, neutral and sad expressions) | 28 right-handed first-time mothers (5 to 10 months postpartum) | Yes |
| **Strathearn et al. (2009)** | Visual (static images) | 3T, 2s events | Photographs of own infant (7 months old) | | Photographs of unknown infants (7 months old) | 30 securely or insecurely attached right-handed first-time mothers (7 months postpartum) | No |
| **Barrett et al. (2012)** | Visual (static images) | 3T, 36s blocks, 3s events | Photographs of own infant (3 months old; happy and sad expressions) | | Photographs of unknown infant (3 months old; happy and sad expressions) | 22 healthy right-handed mothers (3 months postpartum) | Yes |
| **Ranote et al. (2004)** | Visual (videos) | 1.5T, 2--40s blocks | Videos of own and unknown infants (4 to 8 months old) | | Traffic videos; videos of unknown infants (4 to 8 months old) | 10 mothers (4 to 8 months postpartum) | Yes |
| **Noriuchi et al. (2008)** | Visual (videos) | 1.5T, 32s blocks | Videos of own child (16 months old) | | Videos of unknown children (16 months old) | 13 right-handed mothers (16 months postpartum) | Yes |
| **Atzil et al. (2011)** | Visual (videos) | 3T, 2min blocks | Videos showing own infants both by themselves and interacting with their mother (4 to 6 months old) | | Videos of unknown infants (both alone and with their mothers; 4 to 6 months old) | 23 mothers (4 to 6 months postpartum) | Yes |
| **Wan et al. (2014)** | Visual (videos) | 1.5T, 30s blocks | Videos of own and unknown infants (4 to 9 months old) | | Traffic videos; videos of unknown infants (4 to 9 months old) | 20 mothers (4 to 9 months postpartum) | Yes |
| **Abel et al. (2017)** | Visual (videos) | 3T, 20s blocks | Videos of own and unknown infants (2 to 10 months old) | | Traffic videos; videos of unknown infants (2 to 10 months old) | 14 mothers (2 to 10 months postpartum); 15 non-mothers | Yes |

***Supplementary table 2: Maternal brain responses to visual stimuli of infants using static pictures***

| **Author (year)** | Strathearn & McClure (2002) | Swain et al. (2003) | | Nitschke et al. (2004) | | Lenzi et al. (2008) | | Strathearn et al. (2008) | | Strathearn et al. (2009) | | | Barrett et al. (2012) | |
| --- | --- | --- | --- | --- | --- | --- | --- | --- | --- | --- | --- | --- | --- | --- |
| **Number and type of participants** | N=8 | N=9-14 | | N=6 | | N=16 | | N=28 | | N=30 | | | N=22 | |
| **Age of infants at time of scan** | 3 – 18 months | Time 1: 2 – 4 weeks  Time 2: 3 – 4 months | | 2 – 4 months | | 6 - 12 months | | 5 – 10 months | | 7 months | | | 3 months | |
| **Study design** | 3T, 6s events | 3T, 30s blocks | | 1.5T, 30s blocks | | 3T, 2 s mini-blocks | | 3T, 2s events | | 3T, 2s events | | | 3T, 36s blocks, 3s events | |
| **Infant visuals and contrast used** | Photos of own infant > unknown infant | Photos of own infant > unknown infant | Photos of infants > neutral control (house) | Photos of own children > unknown children | Correlation with positive mood | Photos of own infant > unknown infant | Emotional infant expressions > neutral emotional expressions | Photos of own versus unknown children in happy, sad and neutral moods | Photos of own versus unknown happy infants | Securely versus insecurely attached mothers, own vs unknown child in happy, sad and neutral moods | Securely versus insecurely attached mothers (happy own infant faces) | Securely versus insecurely attached mothers (sad own infant faces) | Photos of own infant > unknown infant (happy faces) | Photos of  own infant  > unknown  infant  (sad faces) |
| **Septal regions (MPOA/ VBNST/caudate head)** |  | ACT |  |  |  |  |  |  |  |  |  |  |  |  |
| **Midbrain (including periaqueductal grey):** |  | ACT | ACT |  |  |  |  | ACT | ACT |  |  |  |  |  |
| **Hypothalamus** |  | ACT |  |  |  |  |  | ACT |  | ACT |  |  |  |  |
| **Thalamus** | ACT | ACT | ACT |  |  |  |  | ACT | ACT |  |  |  | ACT |  |
| **Limbic structures:** |  |  |  |  |  |  |  |  |  |  |  |  |  |  |
| Amygdala |  |  | ACT |  |  | ACT | ACT |  | ACT |  |  |  | ACT |  |
| Anterior cingulate |  | ACT | ACT |  |  |  |  |  |  |  |  |  |  | ACT |
| Middle cingulate |  | ACT | ACT |  |  |  |  | ACT |  |  |  |  |  |  |
| Posterior cingulate |  |  |  |  |  |  |  |  |  |  |  |  |  |  |
| Anterior paracingulate |  |  |  |  |  |  |  |  |  |  |  |  |  |  |
| Hippocampus | ACT |  |  |  |  |  |  |  |  |  |  |  |  |  |
| **Basal ganglia:** |  |  |  |  |  |  |  |  |  |  |  |  |  |  |
| Striatum/putamen/  nucleus accumbens | ACT |  | ACT |  |  |  |  | ACT | ACT |  | ACT | ACT |  | ACT |
| Lentiform nucleus  Globus pallidus | ACT |  | ACT |  |  |  |  |  |  |  |  |  |  |  |
| **Insula** |  |  |  |  |  | ACT | ACT | ACT |  | ACT |  |  |  |  |
| **Frontal cortex:** |  |  |  |  |  |  |  |  |  |  |  |  |  |  |
| Orbitofrontal/Inferior  frontal gyrus |  | ACT | ACT | ACT | COR | ACT |  | ACT |  |  | ACT | ACT |  |  |
| Medial frontal gyrus |  |  | ACT |  |  |  |  |  |  | ACT | ACT |  |  | ACT |
| Superior frontal gyrus |  |  |  |  |  |  |  | ACT |  | ACT |  | ACT |  |  |
| Dorsolateral prefrontal cortex |  |  |  |  |  |  |  |  |  |  |  |  |  |  |
| Ventral prefrontal cortex |  |  |  |  |  | ACT |  |  |  |  |  |  |  |  |
| Precentral gyrus |  |  |  |  |  |  |  | ACT |  |  |  |  |  |  |
| Gyrus rectus |  |  |  |  |  |  |  |  |  |  |  |  |  |  |
| **Temporal/parietal cortex:** |  |  |  |  |  |  |  |  |  |  |  |  |  |  |
| Post central gyrus |  |  |  |  |  |  |  |  |  |  |  |  |  | ACT |
| Temperoparietal cortex |  | ACT | ACT |  |  | ACT | ACT | ACT |  |  |  |  |  |  |
| Fusiform gyrus | ACT | ACT | ACT |  |  |  |  | ACT |  |  |  |  |  |  |
| Temporal/auditory cortex |  |  |  | ACT |  | ACT | ACT |  |  |  |  |  | ACT | ACT |
| **Parahippocampal/limbic lobe** |  |  | ACT |  |  |  |  | ACT |  |  |  |  |  |  |
| **Occipital cortex** | ACT | ACT | ACT | ACT |  |  |  | ACT |  |  |  |  |  |  |
| **Cerebellum** | ACT | ACT | ACT | ACT |  |  |  | ACT |  |  |  |  | ACT | ACT |

*ACT= activated; COR= correlated; based on Swain et al. (2007)*

***Supplementary table 3: Maternal brain responses to visual stimuli of infants using video clips***

| **Author (year)** | Ranote et al. (2004) | | Noriuchi et al. (2008) | Atzil et al (2011) | | Wan et al. (2014) | | Abel et al. (2016) US | |
| --- | --- | --- | --- | --- | --- | --- | --- | --- | --- |
| **Number and type of participants** | N=10 | | N=13 | N=23 | | N=20 | | N =14 | |
| **Age of infants at time of scan** | 4 – 8 months | | 16 months | 4 - 6 months | | 4 – 9 months | | 2 – 10 months | |
| **Study design** | 1.5T, 20 – 40s blocks | | 1.5T, 32s blocks | 3T, 2min blocks | | 1.5T, 30s blocks | | 3T, 20s blocks | |
| **Infant visuals and contrast used** | Silent video clips of infants > videos of traffic (control) | Silent video clips of own children > silent video clips of unknown children | Silent video clips of own children > silent video clips of unknown children | Silent video clips of own infants both playing individually and interacting with their mothers > Silent video clips of unknown infants both playing individually and interacting with their mothers | Synchronous mothers (own > other infant) > Intrusive mothers (own > other infant) | Silent video clips of infants > videos of traffic (control) | Silent video clips of own children > silent video clips of unknown children | Silent video clips of infants > videos of traffic (control) | Silent video clips of own children > silent video clips of unknown children |
| **Septal regions (MPOA/ VBNST/caudate head)** |  |  |  | ACT | ACT |  |  |  |  |
| **Midbrain (including periaqueductal grey)** |  |  | ACT |  |  |  |  |  |  |
| **Hypothalamus** |  |  | ACT |  |  |  |  |  |  |
| **Thalamus** |  |  | ACT | ACT |  |  |  |  |  |
| **Limbic structures:** |  |  |  |  |  |  |  |  |  |
| Amygdala |  | ACT |  | ACT |  |  | ACT |  |  |
| Anterior cingulate |  |  |  |  |  | ACT |  |  |  |
| Middle cingulate |  |  |  |  |  |  |  |  |  |
| Posterior cingulate |  |  | ACT |  |  |  |  |  |  |
| Hippocampus |  |  |  |  |  |  |  | ACT |  |
| **Basal ganglia:** |  |  |  |  |  |  |  |  |  |
| Striatum/putamen/  nucleus accumbens |  |  | ACT | ACT |  |  |  |  |  |
| Lentiform nucleus  Globus pallidus |  |  |  |  |  |  |  |  |  |
| **Insula** |  |  | ACT | ACT | ACT | ACT |  |  |  |
| **Frontal cortex:** |  |  |  |  |  |  |  |  |  |
| Orbitofrontal/Inferior  frontal gyrus |  |  | ACT |  | ACT | ACT |  | ACT | ACT |
| Medial frontal gyrus |  |  | ACT | ACT | ACT | ACT | ACT | ACT | ACT |
| Ventral prefrontal  cortex |  |  |  |  |  |  |  |  |  |
| Dorsomedial prefrontal  cortex |  |  | ACT |  |  |  |  |  | ACT |
| Precentral gyrus |  |  | ACT |  |  |  |  | ACT | ACT |
| **Temporal/parietal cortex:** |  |  |  |  |  |  |  |  |  |
| Post central gyrus |  |  |  |  |  |  |  |  |  |
| Temperoparietal cortex | ACT |  |  | ACT |  | ACT | ACT | ACT |  |
| Fusiform gyrus |  |  |  |  |  | ACT |  | ACT |  |
| Temporal/auditory  cortex |  | ACT | ACT |  | ACT | ACT |  | ACT |  |
| **Parahippocampal/limbic lobe** |  |  |  |  |  | ACT |  |  |  |
| **Occipital cortex** | ACT | ACT |  |  |  | ACT | ACT |  |  |
| **Cerebellum** | ACT |  |  | ACT |  | ACT | ACT | ACT |  |

*ACT= activated; based on Swain et al. (2007)*

***Supplementary table 4: Meta-analysis of maternal brain activation to images of own versus control children^a^ (P=0.0001)***

| **Coordinates** | | |  |  | **Volume** | **Maximum** |
| --- | --- | --- | --- | --- | --- | --- |
| **x** | **y** | **z** | **Hemisphere^+^** | **Label^++^** | **(mm3)** | **ALE value** |
| -10 | -8 | 6 | L | Thalamus (Ventral Lateral Nucleus) | 224 | 0.0186 |
| 44 | -18 | 38 | R | Precentral Gyrus (BA 4) | 144 | 0.0168 |
| -22 | 2 | 16 | L | Limbic Lobe, including Uncus (BA 34) and Amygdala | 40 | 0.0134 |
| -12 | 2 | 16 | L | Caudate | 40 | 0.0150 |
| -44 | -16 | 38 | L | Precentral Gyrus (BA 4) | 16 | 0.0138 |

^a^ Significant clusters uncorrected p*≤*0.0001, no cluster extent threshold. **^+^** L=Left; R=Right; **^++^** BA=Brodmann Area. All coordinates in Talairach space.
